# The Treatment of Patients with Early-Stage Non-Small Cell Lung Cancer Who Are Not Candidates or Decline Surgical Resection: The Role of Radiation and Image-Guided Thermal Ablation

**DOI:** 10.3390/jcm13247777

**Published:** 2024-12-19

**Authors:** David S. Buchberger, Rishabh Khurana, Michael Bolen, Gregory M. M. Videtic

**Affiliations:** 1 Department of Radiation Oncology, Taussig Cancer Institute, Cleveland Clinic, Cleveland, OH 44195, USA; buchbed2@ccf.org; 2 Department of Diagnostic Radiology, Cleveland Clinic, Cleveland, OH 44195, USA; khuranr2@ccf.org (R.K.); bolenm@ccf.org (M.B.)

**Keywords:** non-small cell lung cancer, early stage, inoperable, radiotherapy, ablation

## Abstract

The standard of care for early-stage NSCLC has historically been surgical resection. Given the association of lung cancer with smoking, a large number of early-stage patients also have active smoking-related medical comorbidities such as COPD precluding surgery. The current approach for treating such inoperable patients is frequently considered to be stereotactic body radiation therapy (SBRT). SBRT (also known as stereotactic ablative radiation therapy or SABR) is a curative modality that precisely delivers very high dose radiation in few (typically <5) sessions. That said, because of their minimal invasiveness and repeatable nature, image-guided thermal ablation therapies such as radiofrequency ablation (RFA), microwave ablation (MWA), and cryoablation (CA) have also been used to treat early-stage lung tumors. For those patients deemed to have “high operative risk” (i.e., those who cannot tolerate lobectomy, but are candidates for sublobar resection), the appropriateness of potential alternatives [e.g., SBRT; ablation] to surgery is an active area of investigation. In the absence of completed randomized phase III trials, the approach to comparing outcomes between surgery, SBRT, or ablative therapies by their efficacy or equivalence is complex. An overview of the role of SBRT and other non-surgical modalities in the management of early-stage lung cancer is the subject of the present review.

## 1. Introduction

Worldwide, lung cancer remains a significant source of cancer diagnoses and deaths [1]. In the United States, there will be an estimated 234,580 new cases and 125,070 deaths in 2024 [2]. Non-small cell lung cancer histology accounts for the majority of lung cancer diagnoses [3]. Since lung cancer screening was first recommended in 2013, the number of early-stage diagnoses has generally increased. From 2010 to 2019, the number of lung cancer cases diagnosed at a localized-only stage rose among men (4.9%) and women (4.5%) [2]. In 2020, 25.5% of lung cancer diagnoses in men were localized-only diagnoses, while local-only disease was diagnosed in 30.6% of women [2]. Taken together, these data continue to suggest that early-stage diagnoses are increasing.

For the operable early-stage NSCLC patient, surgical resection is the standard of care [4]. For the inoperable patient, the current standard is often now considered to be stereotactic body radiation therapy (SBRT). SBRT (also known as stereotactic ablative radiation therapy or SABR) is defined as very high dose, meticulously targeted radiation given over ≤5 treatments using sophisticated accuracy ensuring techniques including motion management, immobilization, and image guidance. That said, because of their minimal invasiveness and repeatable nature, image-guided thermal ablation therapies such as radiofrequency ablation (RFA), microwave ablation (MWA), and cryoablation (CA) have also been used to treat lung tumors, particularly in patients with contraindications to surgery. For patients deemed to have “high operative risk” (i.e., those who cannot tolerate lobectomy, but are candidates for sublobar resection) stage I NSCLC, the appropriateness of potential alternatives [e.g., SBRT; ablation] to surgery is an active area of investigation. In the absence of completed randomized phase III trials, the approach to comparing outcomes between surgery, SBRT, or ablative therapies by their efficacy or equivalence is complex and has been driven by retrospective reviews and institutional reports. This does make conclusions from these studies subject to bias given the impact that patient, tumor, and treatment factors such as imbalances in the burden of medical comorbidity and lack of congruence in disease staging may have when treatments are being selected. In acknowledging this reality, non-randomized studies become, by necessity, the primary source for evidence-based comparisons between surgical and non-surgical approaches in the management of early-stage lung cancer. An overview of the role of SBRT and other non-surgical modalities in the management of early-stage lung cancer is the subject of the present review (Table 1).

## 2. The Origins and Evolution of Lung SBRT

### 2.1. SBRT for the Inoperable Patient: Origins

The origins of SBRT for early-stage NSCLC lie in the medical complexity of the inoperable patient. Considering the factors that contribute to the development of lung cancer, it is no surprise that a notable proportion of patients presenting with lung cancer have comorbid conditions that exclude them from surgical intervention [6]. Historically, when patients were not candidates for surgery, their disease was managed with external beam radiotherapy (EBRT). Retrospective studies analyzing EBRT alone for the treatment of early-stage NSCLC suggested it yielded inferior survival and cancer control when compared with surgery [7,8,9]. Such poorer outcomes reflected both the impact of the impaired population being selected for EBRT, as well as the technical limitations inherent to EBRT at the time. The latter included such issues as difficulties in accurate target delineation, inadequacy of clinical staging, and incorporation of large volumes of normal lung in the target. From these historical studies it became clear that to improve outcomes with radiotherapy, a new approach was necessary [10].

It was theorized that increasing the biologically equivalent dose (BED) of the delivered radiation could enhance response. BED is a radiobiological quantity that combines the total dose, dose per fraction, and the alpha/beta ratio (a value representing the inherent radiosensitivity of a tumor or tissue) to derive the “true dose” delivered from a given treatment [10,11]. Higher doses per fraction, delivered over shorter time intervals, have higher BEDs. The clinical application of this concept was pioneered by Lars Leksell in Sweden in the 1950s. At that time, Leksell successfully showed that high doses of radiation could be delivered safely and precisely to intracranial lesions using a stereotactic headframe and sophisticated localization system. This method of radiotherapy delivery became known as stereotactic radiosurgery (SRS) and its application to intracranial lesions became routine [12].

**Figure 1:**
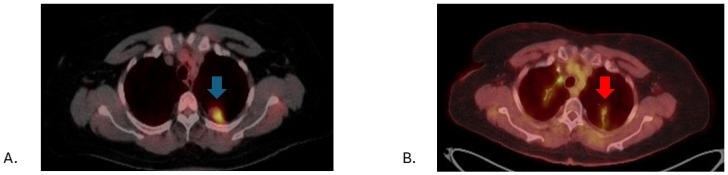
Representative fused PET/CT axial imaging slices from a 68-year-old female with medically inoperable, early-stage squamous cell carcinoma of the left upper lobe lung, pre- and post-lung stereotactic body radiotherapy (SBRT); 34 Gy in 1 fraction on 12/21/2020. (**A**) Lung cancer with SUVmax of 15.3 (blue arrow) pre-SBRT on 11/18/2010. (**B**) Lung cancer with SUVmax of 3.6 (red arrow) post-SBRT on 08/14/2017.

In the 1990s, from the same group in Sweden that pioneered SRS, this concept was applied for the first time to lesions outside of the CNS. Their initial reports described the use of a stereotactic body frame and fixation system to rigidly but non-invasively immobilize patients for accurate high dose treatments, and the technique became known as stereotactic body radiation therapy or SBRT [13]. The low rates of toxicity and high rates of control observed in these initial investigations triggered widespread interest in this approach, and SBRT was rapidly introduced into the early-stage NSCLC treatment armamentarium [13,14] (Figure 1).

### 2.2. SBRT for the Inoperable Patient: Landmark Data, Quality of Life, and Public Health Benefits

After the landmark 1994 publication from the Karolinska Institute in Sweden, a vast body of evidence from a multitude of institutional experiences and numerous non-randomized trials emerged from all around the world showing that lung SBRT results in impressively high rates of local control with minimal treatment-related toxicity [6,7,15,16,17,18,19,20,21,22,23,24,25,26,27,28,29,30,31,32,33,34,35,36,37,38,39,40,41] (Table 2). Throughout the early 2000s, a number of innovative studies from Japan and North America were published showing that high doses per fraction were reproducibly safe and feasible with high rates of control (typically >90%) and low rates of grade 3 or greater toxicity (typically <10%) [22,23,25,33]. Other general insights from these early studies included the observation that central tumors (with centrality defined as tumor within 2 cm of the proximal bronchial tree) had a defined dose- and location-dependent safety profile compared to peripheral tumors and that the higher the BED, the higher the local control [22,23,42].

The publication of the Radiation Therapy Oncology Group (RTOG) trial #0236 in 2010 was a landmark in that regard. RTOG #0236 was the first North American multi-institutional phase II study looking at the safety and efficacy of SBRT in early-stage inoperable NSCLC [26]. In this trial, 55 medically inoperable patients with peripherally located lung tumors were treated to a dose of 54 Gy in three fractions. Five-year local control was 92.7% and there was a 27.3% grade 3 toxicity rate at extended follow-up [26,27]. A subsequent trial, RTOG #0915, investigated single-fraction radiotherapy (34 Gy in 1 fraction) versus multi-fraction regimens (48 Gy in 4 fractions) in the treatment of peripheral NSCLC lesions in inoperable patients [16]. Results first published in 2015 showed toxicity rates between 10% and 15% and local control rates of 92% to 97%. Long-term retrospective data on single-fraction SBRT from this study shows such results to be sustainable over time [17,20].

The increased toxicity observed with centrally located tumors (within 2 cm of the proximal bronchial tree) in some early studies led to additional investigations, including RTOG #0813, a prospective phase I/II trial studying the maximally tolerated dose for centrally located NSCLC lesions treated with SBRT [24]. RTOG 0813 concluded that the MTD for central tumors was 12 Gy per fraction, with acceptable toxicity and similar rates of control and survival between the 10 Gy and 12 Gy per fraction cohorts, subsequently setting the dosing standards used for these tumors today. Despite this, it was observed that for “ultracentral” tumors—initially defined as those within 1 cm of the proximal bronchial tree or invading the mainstem bronchi or trachea—the risk of severe toxicity was still significantly higher, with early Scandinavian studies investigating the treatment of such lesions having grade 5 toxicity rates on the order of 20% [45,46]. Recently, the Stereotactic Radiation for Ultra-Central Non-Small Cell Lung Cancer: A Safety and Efficacy Trial (SUNSET) was published [43]. In this study, ultracentral tumors were defined as those with PTV abutment with the proximal bronchial tree, esophagus, pulmonary vein, and/or pulmonary artery, and rigorous treatment planning techniques were employed. Patients were treated to a dose of 60 Gy in eight fractions. Thirty were enrolled, with two grade 3 to grade 5 adverse events (grade 3 dyspnea, grade 5 pneumonia in the setting of ILD). Three-year local control was 89.6% and there was no clinically or statistically significant decline in QOL scores. While these results are encouraging, the optimal dose and fractionation schedule for the treatment of ultracentral tumors remains an area of active investigation [32,36,47,48,49,50].

SBRT has been directly compared with conventional EBRT in the treatment of early-stage NSCLC in inoperable patients in two randomized controlled trials. In 2016, the Stereotactic Precision And Conventional radiotherapy Evaluation (SPACE) trial was published, ultimately finding no difference between the SBRT or conventionally fractionated arms in terms of progression-free survival, overall survival, or local control, with better quality of life outcomes in the SBRT group [30]. TROG 09.02 CHISEL was a phase III randomized trial that also compared SBRT to conventional EBRT. At a median follow-up of over two years, CHISEL demonstrated statistically significant improvements in local control and overall survival compared to the conventional EBRT group, with no major differences in toxicity, confirming SBRT as superior to conventionally fractionated RT in this population and establishing it as the standard of care [31]. More recently, SBRT has been compared to hypofractionated RT in the phase III Canadian LUSTRE trial. While the trail failed to meet accrual, there was no difference in LC, EFS, OS, or toxicity between the two arms, giving further credibility to the efficacy and convenience of SBRT for the treatment of early-stage NSCLC relative to other RT regimens [44].

In addition to the above data regarding the safety and efficacy of SBRT, numerous publications have highlighted the favorable quality of life (QOL) benefits of SBRT [51,52,53]. One notable example is a 2018 phase II trial that investigated the early impact of SBRT on QOL, finding that SBRT resulted in an early and significant improvement of QOL scores for those with low pre-treatment scores [53]. Similarly, a systematic review of nine early-stage lung cancer studies analyzing the association between receipt of SBRT and QOL showed that SBRT was not a detriment to QOL over numerous domains [52]. Lung SBRT is also highly cost-effective. Studies using Markov modeling suggest that among alternatives including standard EBRT and radiofrequency ablation, lung SBRT is the most cost-effective by a significant margin. In one study, the incremental cost-effectiveness of SBRT compared to conventionally fractionated RT was USD 6000 per quality-adjusted life year, and USD 14,100 per quality-adjusted life year compared to RFA [54].

While early studies excluded large tumors (>5 cm) and use in salvage situations, subsequent work has suggested that SBRT can be efficacious in these settings as well. In the initial publication assessing SBRT in tumors > 5 cm (median: 5.6 cm, range: 5.1–10 cm), an 18-month local control rate of 91.2% was reported with a 7.5% rate of grade 3 or greater toxicity [10,21]. Subsequent publications have found similar results, suggesting a role for SBRT in the treatment of larger tumors [55]. The use of SBRT has also been explored in the salvage setting. In 2014, Hearn et al. described the successful use of salvage SBRT for 2022 patients after local recurrence following previous SBRT [38]. The same group also published a series on 48 patients with local failure after surgical resection treated successfully with SBRT [29]. With respect to tumor histology, some studies have suggested that adenocarcinoma has higher rates of local control compared to squamous cell carcinoma when treated with fractionated SBRT schedules, while others have failed to show a difference in outcome based on histology [56,57]. Of note, histology does not seem to be a factor affecting control when treating with SF-SBRT [20].

### 2.3. SBRT in the Operable Patient

Building on early work from Japanese researchers that showed a 5-year OS rate of 70.8% for operable patients treated with SBRT doses corresponding to a BED ≥ 100 [22,23], various parties eventually endeavored to compare SBRT directly to surgery. However, poor accrual resulted in very low completion rates for these comparison studies. Pooled analyses of failed trials have suggested a benefit of SBRT over surgery, but such studies are controversial and have been highly critiqued [58,59]. Direct comparisons aside, SBRT in the operable population has been investigated in the phase 2 setting. In 2018, RTOG #0618 was published by Timmerman et al., a cooperative group trial evaluating the use of SBRT in operable early-stage NSCLC with surgery reserved for salvage in the event of local failures [28]. In this study, SBRT was delivered to peripheral lesions as 54 Gy in 3 fractions. While this study did not directly compare SBRT to surgery, it did provide data on the benefit of SBRT in the operable population. The local control rate at 4 years was 96%. The one local failure was successfully salvaged with surgery.

A number of phase III trials are addressing the question of surgery versus SBRT for the early-stage operable NSCLC patient. These include the Sublobar Resection (SR) versus Stereotactic Ablative Radiotherapy (SAbR) in High-Risk Patients with Stage I Non-Small Cell Lung Cancer (NSCLC) (STABLEMATES) [60] as well as the Veterans Affairs Lung Cancer Surgery Or Stereotactic Radiotherapy (VALOR) trial [61] and the SORT Trial (Comparing the Effectiveness of Surgery versus Stereotactic Body Radiation Therapy for Stage I Non-Small Cell Lung Cancer) [62].

### 2.4. Adjuvant Therapy

It has been shown that the primary mode of failure after SBRT is distant metastasis [63,64]. Considering the exceptional local control rates of SBRT (consistently on the order of 90%), the prevention of distant failure is essential for optimizing outcomes. Achieving distant control has been historically challenging, as the frailty of the SBRT population often precluded prolonged use of conventional chemotherapy. The advent of immunotherapy has revolutionized the treatment of lung cancer, and its favorable toxicity profile has opened up a potential means to distant control not previously available in this population [65,66,67,68,69,70,71,72,73]. In 2023, MD Anderson published the results of a phase II trial comparing SBRT alone versus SBRT plus immunotherapy (nivolumab) in the treatment of patients with early-stage NSCLC. Those patients receiving SBRT with immunotherapy had significantly improved 4-year event-free survival (77% vs. 53%, *p* = 0.0056), with improvements across the board in local, regional, and distant failure [74].

Multiple on-going trials are actively investigating the integration of SBRT and immunotherapy. These trials include PACIFIC-4, an international phase III study comparing SBRT alone to SBRT with concurrent and adjuvant durvalumab [75], and KEYNOTE-867, a phase III randomized trial comparing SBRT alone to SBRT plus concurrent and adjuvant pembrolizumab in early-stage NSCLC [76].

## 3. Image-Guided Thermal Ablation (IGTA) Procedures

### 3.1. Introduction

Percutaneous IGTA employs either extreme heat or cold to destroy tumor cells and serves as a well-tolerated, cost-effective, and minimally invasive treatment option. It is particularly suitable for medically inoperable patients or those considered high-risk surgical candidates. It can be used as a standalone curative modality, a palliative treatment, or as an adjunct to surgery, chemotherapy, or radiation [77]. Furthermore, as lung cancer screening has enabled earlier detection of premalignant or early-stage locoregional tumors, minimally invasive treatments are increasingly utilized [78,79,80]. IGTA offers the additional advantage of allowing for simultaneous biopsy collection as part of the image-guided procedure, along with the benefit of minimized collateral damage to adjacent tissues.

### 3.2. IGTA Modalities and Mechanism of Action

Percutaneous thermal ablation encompasses both hyperthermic techniques, such as radiofrequency and microwave ablation, and hypothermic techniques like cryoablation. These procedures are performed under image guidance, with Computed Tomography (CT) being the primary modality, which may be conventional CT (CCT), CT Fluoroscopy (CTF), or Cone Beam CT (CBCT). In certain cases, ultrasound (USG) may also be used alongside CT [78,81].

Radiofrequency ablation (RFA) has been the most extensively studied modality. An active RF electrode is placed within the tumor using image guidance. A grounding pad (reference electrode) is applied to the opposite chest wall or thigh region. The RF electrode generates radiofrequency waves in the range of 375–500 kHz. These generate electric field lines between the electrodes, which oscillate with the alternating current. The energy disperses into the surrounding tissues and exits through the ground pads. Molecular collisions adjacent to the RF electrode generate frictional heat, with local temperatures typically rising above 55 degrees Celsius, which ultimately leads to cell cytotoxicity and protein denaturation, which induces coagulative necrosis. For larger lesions, multiple ablations may be facilitated either by probe repositioning or using multiple applicators. As surrounding normal lung tissue is a poor conductor of heat and electric current, the generated heat concentrates within the tumor. Care must be taken to keep the electrode tip temperature below 100 degrees Celsius, to prevent charring and vaporization, as these will reduce the effectiveness of ablation by altering electrical impedance and heat dissipation. The limitations include “heat-sink phenomenon” due to transfer of heat to nearby vasculature, non-visualization of the ablation zone during the procedure, and the possibility of skin burns at the site of grounding electrodes [82,83,84,85,86,87].

**Figure 2:**
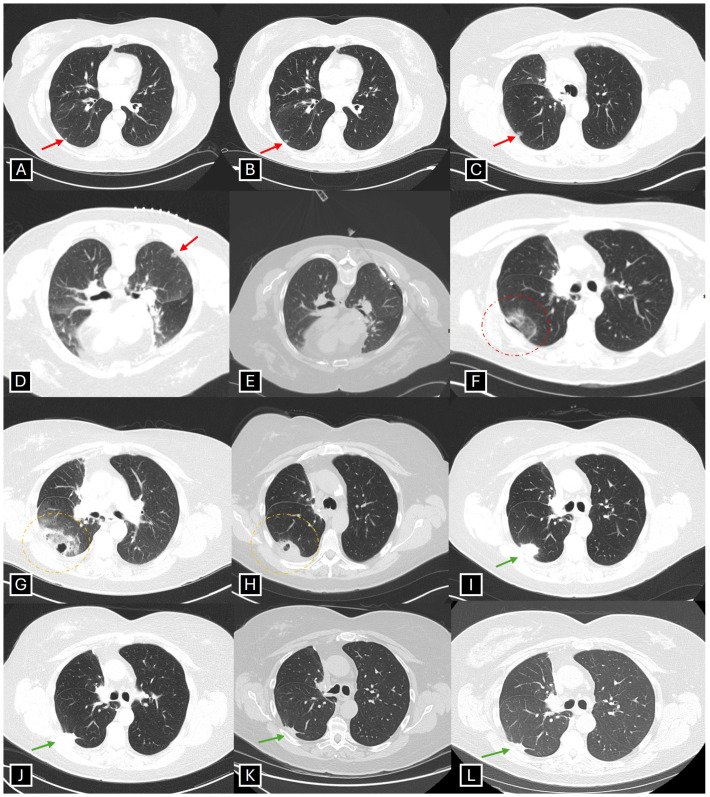
An elderly woman, former smoker, with prior right upper lobectomy for lung cancer, had a suspicious progressively enlarging (over three years) right lower lobe part-solid nodule concerning for lesion along adenocarcinoma spectrum (red arrow, **A**–**C**). (**D**) Patient in prone position with grid lines in place for nodule localization. (**E**) Microwave ablation procedure with the antenna within the nodule. (A single CT guided biopsy was obtained prior to microwave ablation procedure which was evaluated by pathology). (**F**) Patient in supine position for post-procedure check CT, depicting trace right-sided pneumothorax and perilesional ground glass opacities consistent with expected perilesional hemorrhage (red circle). (**G**) Post-procedure Day 1 CT showing ground glass opacity with peripheral consolidation, and central cavitation, consistent with post ablation changes (orange circle). (**H**) Dense consolidative opacity with decrease in size of central cavitation in keeping with evolving post-ablation changes (orange circle). (**I**–**L**): Sequential follow up CTs at 6 months, 1 year, 4 year, and 5 years, respectively, in keeping with stable post-ablation changes (green arrow) without imaging evidence of local recurrence.

Microwave ablation (MWA) has been gaining popularity in the treatment of lung tumors. It utilizes radiation in the microwave energy range (300–3000 MHz), creating an oscillating electromagnetic field which increases kinetic energy of water molecules, generating frictional heat leading to coagulative necrosis [78,87]. Unlike RFA, MWA has a better convection profile, which is less reliant on heat conduction, thereby creating larger ablation zones (even in presence of charred tissue), reduces the “heat-sink phenomenon”, has relatively faster ablation time, and creates a more uniform ablation zone. Additionally, the risk of associated skin burns is decreased, as grounding pads are not required [84,85,88,89]. Figure 2 depicts the results of a CT-guided MWA procedure performed for an early-stage NSCLC.

Cryoablation (CA) utilizes the Joule–Thompson effect, in which gases (for example argon, nitrogen, and nitrous oxide) experience a temperature drop upon moving from high to low pressure. Subzero temperatures or alternating freeze–thaw cycles lead to ice crystal formation, protein denaturation, microvascular thrombosis, and osmotic shifts, ultimately producing coagulative necrosis and cell membrane lysis. The temperature can be taken down as low as −170 degree Celsius near the needle tip, and gradually becomes warmer towards the peripheral edges. The target temperature is typically around −40 degree Celsius (cell death generally occurs around −20 degree Celsius). The effect can be enhanced by using multiple probes, variable needle sizes/shapes, and freeze–thaw cycles. CA allows real-time optimization as ice balls and ablation zone are visible during the procedure. CA specifically preserves the structures with a collagenous matrix, such as blood vessels and bronchial airways, making it an ideal option for treating tumors located near major blood vessels and the hilum. Further, it induces less pain as compared to other heat-based modalities. Some drawbacks include variable ablation sizes, extended ablation times, the associated risk for cryo-shock, and greater procedural complexity [78,81,82,87,90,91,92].

### 3.3. Indications/Patient Selection: Society Guidelines for IGTA for Early NSCLC

The choice of energy modality should consider tumor size, location, complication risks, and local expertise. Several clinical practice guidelines from various professional societies provide recommendations on the use of IGTA for NSCLC [93]. The National Comprehensive Cancer Network (NCCN) suggests IGTA as a treatment option for “high-risk” patients, or those whose tumors are surgically resectable but deemed medically inoperable due to patient comorbidities. They recommend a multidisciplinary approach and affirm IGTA for NSCLC tumors under 3 cm size, multiple primary lung cancers where local therapy is feasible, as well as locoregional recurrences [94]. The Cardiovascular and Interventional Radiology Society of Europe (CIRSE) recommends IGTA for patients with primary lung cancer who are unsuitable for surgery. Better results are on record for lesions ≤2 cm and at least 1 cm tumor margin ablation. Although the efficacy of MWA is comparable to RFA, MWA is more suitable for larger lesions. Moreover, CIRSE also stresses the importance of using pre-procedural contrast-enhanced CT (CECT) and FDG-PET/CT for accurate staging, and pulmonary function tests for those with a history of lung disease or surgery [93]. The Society of Interventional Radiology (SIR), in a multidisciplinary position statement endorsed by other societies, supports IGTA as a viable treatment for inoperable stage I NSCLC [93]. While no direct comparisons between the different IGTA techniques have been conducted, the available indirect evidence suggests that all three are viable options for lung ablation. Each modality comes with its own set of advantages and disadvantages, which should be carefully evaluated while making treatment decisions [93,95,96,97]. Absolute contraindications include severe lung emphysema with bullae, life expectancy <3 months, ECOG >2, and non-correctable hemorrhagic diathesis. Relative contraindications include impaired lung function, tumors located near large vessels or hilum, and correctable hemorrhagic diathesis [81].

### 3.4. Technique

IGTA is typically performed under CT guidance, with patient positioning determined by factors such as tumor location (central vs. peripheral), available trajectory, and the need to avoid crossing multiple pleurae or intersecting the broncho-vascular bundle. To avoid movement and minimize the risk of pneumothorax, prone positioning is preferred over the lateral decubitus approach. The use of general anesthesia helps control respiratory motion, provides cardiopulmonary support, and improves patient comfort. Local anesthesia should be applied along the skin, needle tract, and pleura to manage pain [85,98]. A detailed comparison of different ablation modalities is covered subsequently.

### 3.5. Safety of IGTA Procedures

Both major and minor complications associated with IGTA for lung malignancy have been evaluated and summarized in registry-based studies, systematic reviews, and meta-analyses [93,97,99,100]. Patients referred for IGTA are often more sick, due to associated comorbidities, versus those referred for other procedures. A systematic review of 34 studies assessing the comparative efficacy and safety of IGTA modalities reported a weighted average major complication rate of 11.5%, with specific rates of 11.6% for RFA, 22.5% for MWA, and 4.6% for CA [97]. It is relevant to note that cryoablation seems to present a lower side-effect profile compared to heat-based ablation techniques. Pneumothorax was the most common complication across all studies and modalities. Other complications include pulmonary hemorrhage, pleural effusion, pneumonia, lung abscess, bronchopleural fistula, and aseptic pleuritis [82,96,97,101,102]. Additionally, a systematic review primarily focused on the safety of RFA revealed pooled major and minor complication rates of 6% (95% CI, 3–8%) and 27% (95% CI, 14–41%), respectively [93,100]. Overall, these low complication rates indicate that ablation is a safe and effective treatment option. In the Society of Interventional Radiology position statement, which has also been endorsed by various other societies (Canadian Association for Interventional Radiology, the Cardiovascular and Interventional Radiological Society of Europe, and the Society of Interventional Oncology), it has been recommended that pneumothorax should be counted as an expected outcome rather than a complication, in future trials [93].

### 3.6. Preservation of Lung Function

Over the years, studies and post hoc analysis of clinical trials have consistently shown that lung function is preserved without permanent decline after IGTA treatment [93,103,104,105]. On the contrary, other forms of local control, for example surgical resection, have been associated with an appreciable reduction in lung function [93,106,107,108].

While a few studies have suggested that ablation causes less decline in lung function relative to SBRT [93,109,110], this must be interpreted in the context of decades of lung SBRT literature that has repeatedly affirmed the safety of SBRT in the early-stage lung cancer population, including in particularly frail groups such as the elderly and those with interstitial lung disease [51,111,112].

Regardless, preserving lung function with less invasive modalities such as IGTA or SBRT is particularly advantageous for patients with comorbid pulmonary insufficiency or those requiring treatment for multiple tumors, whether due to multiple synchronous or metachronous primary cancer. Patients needing multiple procedures for lung tumors are often well-suited for percutaneous ablation techniques since repeated surgery or re-irradiation scenarios can sometimes have limited options.

### 3.7. Comparative Assessment of IGTA Versus Surgical Resection and SBRT

For patients with stage I or II NSCLC, surgical resection remains the most curative option, with 5-year survival rates for stage I and stage II ranging from 60 to 80% and 30 to 50% respectively. However, only one-third of patients are suitable candidates for lobar or sublobar resection [93,113,114]. Although no randomized trials have compared percutaneous IGTA with surgery or SBRT, several non-randomized studies provide insight into their comparative assessment. However, the studies are limited by their retrospective design, inconsistent data reporting, different baseline characteristics between groups, which complicate direct comparisons, and inherent selection bias. Thus, the results may not fully reflect the representative broader population [26,93,105,113].

Registry-based cohort studies and multiple retrospective studies evaluating IGTA versus surgical resection (sublobar resection or lobectomy) have shown comparable overall survival rates and disease-free survival rates, implying similar efficacy [93,106,115,116,117,118,119].

Some studies comparing IGTA versus SBRT have shown no statistical difference between overall survival rates, local tumor progression rates, disease progression, or any cause mortality [93,113,119,120,121], while others have suggested better local control and lower toxicity with SBRT.

A systematic review and pooled analysis published in 2016 evaluated outcomes for over 3000 patients treated across 44 studies. Local control rates at 1, 3, and 5 years were 77%, 55%, and 42% in the RFA group versus 97%, 88%, and 86% in the SBRT group (*p* < 0.001). These differences persisted after accounting for stage and age. However, tumor size (>3 cm) may be a factor favoring SBRT over IGTA [5]. The rate of pneumothorax was 31% in the RFA cohort, while the rate of grade ≥3 radiation pneumonitis was 2%. These differences in control and toxicity did not translate into an overall survival difference [5,93].

### 3.8. IGTA: RFA Versus MWA Versus CA

RFA has been the most extensively studied among all the thermal ablation modalities. The RAPTURE trial (2008) demonstrated the feasibility, safety, and efficacy of RFA for treating lung malignancies. There were no procedure-related deaths or significant declines in pulmonary function. A complete response lasting at least one year was observed in 88% of assessable patients. For patients with NSCLC, the 1-year and 2-year overall survival (OS) rates were 70% and 48%, respectively [104]. A more recent prospective multicenter trial from the American College of Surgeons Oncology Group (Z4033, Alliance) examined the safety and efficacy of RFA in patients with medically inoperable stage IA NSCLC (n = 51). The study reported a 1-year OS of 86.3% and a 2-year OS of 69.8%, with local tumor recurrence-free rates of 68.9% and 59.8% at 1 and 2 years, respectively [105]. Various other studies analyzing the role of RFA include studies by Ambrogi et al., Palussiere et al., and Huang et al. [107,108,122,123].

MWA for stage I NSCLC has been evaluated in few small, single-arm studies. Yang et al. reported survival rates of 89%, 63%, 43%, and 16% at 1, 2, 3, and 5 years, respectively, in patients with stage I NSCLC [124]. Han et al. documented survival rates of 97.1%, 92.6%, 63.4%, 54.4%, and 32.6% at 1, 2, 3, 4, and 5 years in a cohort of 63 patients aged 80 years or older who underwent MWA for stage IA NSCLC [125].

CA has been evaluated in a single-arm prospective trial. Moore et al. reported 5-year outcomes following percutaneous CA for stage I NSCLC in 45 patients with 47 biopsy-proven tumors. The overall survival (OS) rates at 1, 3, and 5 years were 89.4%, 78.1%, and 67.8%, respectively. The 5-year progression-free survival (PFS) and cancer-specific survival (CSS) were 87.9% and 56.6%, respectively [126]. A recently published metanalysis showed superiority of CA over RFA in terms of 3-year disease-free survival, reduced recurrence rates, and complication rates [102]. Recent studies focusing on treatment in the elderly show promising outcomes [127,128]. One study assessing outcomes in octogenarians with stage IA-IIB primary lung malignancies reported a 94% 3-year OS rate and 100% recurrence-free survival throughout the imaging follow-up period [128].

While no robust direct comparisons between the different IGTA techniques have been conducted, the available indirect evidence suggests that all three are viable options for lung ablation (Table 3). Each modality comes with its own set of advantages and disadvantages, which should be carefully evaluated when making treatment decisions [93,95,96,97].

### 3.9. Cost-Effectiveness

Current evidence suggests that the overall cost of care for an early-stage NSCLC is significantly lower for patients treated with IGTA compared to surgical treatment options [93,106,127]. Since IGTA for stage IA NSCLC offers comparable survival outcomes to sublobar resection and SBRT, the cost of healthcare delivery for each intervention may influence the choice of treatment.

### 3.10. IGTA Conclusions

The available evidence for early-stage NSCLC treatment suggests that all above-mentioned image-guided tumor ablation modalities, when used appropriately, can be effective and have amply demonstrated comparable survival outcomes to surgical resection and SBRT, making it a viable treatment option. The choice of energy modality should primarily be guided by lesion characteristics and risk management. Additionally, factors such as local expertise, operator familiarity, ease of use of the device, multi-modality team meetings, physician preferences, and cost should be considered in the overall decision-making process and patient management [93,129].

## Figures and Tables

**Table 1:** Comparison of lung SBRT and IGTA.

Modality	MOA	Delivery	Indications	Outcomes	Toxicity	Comparisons
Lung SBRT	High energy XRAYs (photons) cause DNA damage leading to tumor cell death	Non-invasive No anesthesia Linear accelerator (most commonly) Patient immobilized (body mold with abdominal compression) 4D CT used to track tumor motion throughout respiratory cycle, or breath-hold techniques used to ensure accurate delivery Daily image guidance used to confirm accuracy	Inoperable early-stage NSCLC or operable patients who decline surgery Used to effectively treat peripheral, central, and ultracentral lesions Per societal guidelines, can be used for the treatment of large tumors (≥5 cm) and salvage situations (post-surgery or re-RT) Lung disease, location to hilum/vessels not absolute contraindications Also commonly used in the treatment of lung metastases	LC rates on the order of 85−98% OS benefit over conventional EBRT shown in PRT phase III setting Acceptable outcomes demonstrated in operable patients	G3/4 toxicity on the order of <10% Common toxicities: pneumonitis, esophagitis, chest wall pain G5 toxicity rare—more concerning with ultracentral tumors and mitigated with meticulous treatment planning at centers of expertise Acceptable doses, peripheral tumors: 34 Gy/1 fx, 48 Gy/4 fx, 54 Gy/3 fx Acceptable doses, central/ultracentral tumors: 50 Gy/5 fx, 60 Gy/8 fx	Bi et al., RFA vs. SBRT, Systemic Review and Pooled Analysis (IJROBP 2016) [5] 31 studies and over 3000 patients analyzed LC RFA—1 yr, 3 yr, 5 yr: 77%, 55%, 42% LC SBRT—1 yr, 3 yr, 5 yr: 97%, 88%, 86% (*p* < 0.001) No OS difference Most frequent RFA complication: Pneumothorax (31%) Most frequent SBRT (≥G3) complication: Pneumonitis (2%)
IGTA	Extreme heat or cold generated via radiofrequency waves, microwaves, Joule–Thompson effect leading to tumor cell death RFA, MWA, CA are all options	Minimally invasive Requires anesthesia/sedation Electrodes, probes and/or needles inserted in tumor under image guidance to facilitate effect	Inoperable early-stage NSCLC or operable patients who decline surgery Preferred for tumors ≤ 3 cm Absolute contraindications include emphysema with bullae, life expectancy < 3 months, ECOG > 3, non-correctable hemorrhagic diathesis Tumor location near hilum/vessels is a relative contraindication	LC rates on the order of 42–77% OS comparable to series of surgery and lung SBRT	Major complication rates: 4.6% to 30.0% Most common complication: Pneumothorax (~30%) Other complications: Hemorrhage, effusion, infection, fistula	

Abbreviations: MOA (mechanism of action), RFA (radiofrequency ablation), MWA (microwave ablation), CA (cryoablation), LC (local control), OS (overall survival), Fx (fraction).

**Table 2:** Selected lung SBRT studies.

Year of Publication	Reference	Design	No. of Patients	Population	Histology	Dose	Outcomes	Toxicity	Notes
2010 2018	RTOG #0236 [26,27]	Phase II	55	T1, T2 N0M0 Medically inoperable Peripheral lung tumors	SCC: 31% Adeno: 35% Large cell: 5% NSCLC, NOS: 29%	54 Gy in 3 fractions	LC, 3 yr: 97.6% LC, 5 yr: 92.7% OS, 3 yr: 55.8% OS, 5 yr: 40.0%	G3/4, 3 yr: 16.4% G3/4, 5 yr: 30.9%	First North American multi-institutional phase II study for lung SBRT in early-stage NSCLC
2015 2019	RTOG #0915 [16,17]	Phase II	84	T1, T2 N0M0 Medically inoperable Peripheral lung tumors	SCC: 29.8% Adeno: 58.3% NSCLC, NOS: 11.9%	34 Gy in 1 fraction 48 Gy in 4 fractions	34 Gy, LC, 1 yr: 97.0% 48 Gy, LC, 1 yr: 92.7% 34 Gy, LC, 5 yr: 89.4% 48 Gy, LC, 5 yr: 93.2% 34 Gy, OS, 5 yr: 29.6% 48 Gy, OS, 5 yr: 41.1%	34 Gy, G3 or higher, 1 yr: 10.3% 48 Gy, G3 or higher, 1 yr: 13.3% 34 Gy, G3 or higher, 5 yr: 2.6% 48 Gy, G3 or higher, 5 yr: 11.1%	Established the validity of single-fraction SBRT for the treatment of peripheral lung tumors
2018	RTOG #0618 [28]	Phase II	26	T, T2 N0M0 Medically operable Peripheral lung tumors	SCC: 19.2% Adeno: 46.1% NSCLC, NOS: 34.6%	54 Gy in 3 fractions	LC, 4 yr: 96.0% OS, 4 yr: 56.0%	G3, 4 yr: 7.7% (No G4/5)	Investigated application of SBRT in the medically operable population, showing promising results
2019	RTOG #0813 [24]	Phase I/II	100	T1, T2 N0M0 Medically inoperable Central lung tumors	SCC: 45.0% Adeno: 39.0% Bronchoalveolar: 1.0% NSCLC, NOS: 15.0%	Five fraction, dose escalation, 10–12 Gy per fraction	LC, 10 Gy per fraction, 2 yr: 87.5% LC, 12 Gy per fraction, 2 yr: 87.9% OS, 10 Gy per fraction, 2 yr: 75% OS, 12 Gy per fraction, 2 yr: 72.7%	Probability of dose limiting toxicity at 12 Gy per fraction: 7.2%	Established safe and effective dosing for central tumors as 50 Gy in 5 fractions
2019	CHISEL [31]	Phase III PRT	101	T1-T2a, N0M0 NSCLC Medically inoperable or refused surgery Peripherally located lung tumor	SCC: 37.6% Adeno: 45.5% Large Cell: 2.0% Mixed: 2.0% NSCLC, NOS: 12.9%	SBRT: 54 Gy in 3 fractions or 48 Gy in 4 fractions vs. EBRT: 66 Gy in 33 fractions or 50 Gy in 20 fractions	SBRT, LC, 2 yr: 89% EBRT: LC, 2 yr: 65% SBRT, MS: 5 years EBRT, MS: 3 years	SBRT, G3/G4: 8 events EBRT, G3: 2 events	In a randomized phase III setting, this trial demonstrated the superiority of SBRT in terms of local control over EBRT while also showing an OS benefit with SBRT and low rates of toxicity
2024	SUNSET [43]	Phase I	30	T1-T3 N0M0 NSCLC (≤6 cm) Ultracentral tumor location Planning target volume overlap with CBT, esophagus, PV, PA	SCC: 26.7% Adeno: 33.3% NSCLC, NOS: 40.0%	60 Gy in 8 fractions	LC, 3 yr: 89.6% OS, 3 yr: 72.5%	G3-5: 6.7% 2 patients—1 G3 dyspnea, 1 G5 PNA (ILD)	Showed 60 Gy in 8 fractions to be an effective and safe dose for ultracentral tumors
2024	LUSTRE [44]	Phase III PRT	233	T1-T2a N0M0 Medically inoperable Peripheral, central, or ultracentral tumor location	N/A	SBRT peripheral: 48 Gy in 4 fractions SBRT central or ultracentral: 60 Gy in 8 fractions Non-SBRT: 60 Gy in 15 fractions	SBRT, LC, 3 yr: 87.6% Non-SBRT, LC, 3 yr: 81.2%	SBRT, G3/4: 12.8% SBRT, G5: 1 event Non-SBRT, G3/4: 7%	No difference in LC, EFS, OS or toxicity between SBRT and hypo-fractionated RT

Abbreviations: PRT (prospective randomized trial), SCC (squamous cell carcinoma), Adeno (adenocarcinoma), NOS (not otherwise specified), LC (local control), OS (overall survival), MS (median survival), PNA (pneumonia), ILD (interstitial lung disease). Not applicable (N/A).

**Table 3:** Selected IGTA studies.

Year of Publication	Reference	Design	No. of Patients	Population	Histology	Modality	Outcomes	Toxicity	Notes
2008	RAPTURE [104]	Prospective, single-arm, multicenter	105 (33 NSCLC patients) 137 procedures	Tumors 3.5 cm or smaller in diameter NSCLC and metastases 13 patients were stage I NSCLC—remaining 20 were recurrent or metastatic	SCC: 54.5% Adeno: 39.3% Large cell: 3% Bronchiolar carcinoma: 3%	RFA	LC, 1 yr: 88% OS, 1 yr, NSCLC: 70% OS, 2 yr, NSCLC: 48%	Pneumothorax needing drainage (total): 27/137 procedures, 19.7% Pneumothorax not needing drainage: 28/137, 20.4% Pleural effusion needing drainage: 4/137, 2.9% Pleural effusion not needing drainage: 11/137, 8% Intrapulmonary hemorrhage: 3/137, 2.2%	First prospective, intention to treat, multicenter trial assessing RFA for lung malignancies
2011	Ambrogi et al. [107]	Prospective, single center study	57 80 procedures	Stage I NSCLC Medically inoperable	SCC: 49.2% Adeno: 25.4% NOS: 25.4%	RFA	Median FU of 47 months CR rate: 59.3% Median OS: 33.4 months Cancer specific actuarial survival: 59% at 3 years	Major complication rate: 5% Minor complication rate: 20% Pneumothorax: 4 requiring drainage	CR rate for stage IA: 65.9% CR rate for stage IB: 40% (*p* = 0.01)
2015	Group Z4033 (Alliance) Trial [105]	Prospective multicenter trial	51	Biopsy proven stage IA NSCLC Medically inoperable	SCC: 37.3% Adeno: 47.1% Bronchoalveolar: 2.0% NOS: 13.7%	RFA	LC, 1 yr: 68.9% LC, 2 yr: 59.8% OS, 1 yr: 86.3% OS, 2 yr: 69.8%	G3-5: 11.8% Authors note no G4 to G5 events were attributable to RFA	LC rates worse for tumors >2 cm
2015	Palussiere et al. [108]	Prospective, multicenter trial	87 88 procedures	N0 NSCLC Medically inoperable	SCC: 21% Adeno: 59% Other: 16% NOS: 4%	RFA (82) MWA (5)	LC, 1 yr; 88.5% LC, 2 yr: 81.7% LC, 3 yr: 78.9% OS, 5- yr: 58.1%	Pneumothorax: 44/88, 50% Pneumothorax requiring drainage: 18/88, 20.5% G3/4 brachial plexopathy: 2	On MVA, pathology and tumor size >2 cm were independent prognostic factors for DFS
2018	Huang et al. [123]	RR	50 73 procedures	Stage IA NSCLC Medically inoperable	SCC: 32% Adeno: 50% Other: 18%	RFA	LC, 1 yr: 4% LC, 2 yr: 12% LC, 5 yr: 26% OS, 1 yr: 96% OS, 3 yr: 67.1% OS, 5 yr: 36.3%	Pneumothorax requiring drainage: 4% Low grade fever: 36% Hemoptysis/cough: 22%	Tumor size <2 cm was associated with significantly improved 3-year OS
2014	Yang et al. [124]	RR	47	Stage I NSCLC Medically inoperable	SCC: 27.7% Adeno: 59.6% Other: 12.7%	MWA	LC, 1 yr; 96% LC, 3 yr: 64% LC, 5 yr: 48% OS, 1 yr; 89% OS, 3 yr: 43% OS, 5 yr: 16%	Pneumothorax: 63.8% Hemoptysis: 31.9% Pleural effusion: 34% Infection: 14.9% Bronchopulmonary fistula: 2.1%	Tumors ≤3.5 cm associated with better survival
2015	Moore et al. [126]	RR	45 patients 47 procedures	T1N0M0 NSCLC Medically inoperable	SCC: 19.1% Adeno: 44.7% NOS: 36.2%	CA	LC: 85.1% OS, 5- yr: 67.8% ± 15.3	Major complications: 6.4% 2 cases of hemoptysis Prolonged chest tube placement	Favorable outcomes for CA

Abbreviations: SCC (squamous cell carcinoma), Adeno (adenocarcinoma), NOS (not otherwise specified), LC (local control), OS (overall survival), MS (median survival), CR (complete response), MVA (multivariable analysis).
